# Human and Feline Sporotrichosis in a Reference Center of Southeastern Brazil: Genetic Differentiation, Diversity, and Antifungal Susceptibility of *Sporothrix* Species

**DOI:** 10.3390/jof9080831

**Published:** 2023-08-07

**Authors:** Sarah Santos Gonçalves, Isabela da Cruz Bahiense Rocha, Bruno Carneiro Rediguieri, Jamile Ambrósio de Carvalho, Simone Bravim Maifrede, Wdson Luis Lima Kruschewsky, Aloísio Falqueto, Anderson Messias Rodrigues

**Affiliations:** 1 Infectious Diseases Postgraduate Program, Center for Research in Medical Mycology, Department of Pathology, Federal University of Espírito Santo (UFES), Vitoria 29043900, Brazil; isabelabahiense@yahoo.com (I.d.C.B.R.); brediguieri@gmail.com (B.C.R.); 2 Laboratory of Emerging Fungal Pathogens, Department of Microbiology, Immunology and Parasitology, Discipline of Cellular Biology, Federal University of São Paulo (UNIFESP), São Paulo 04023062, Brazil; jamileambrosio@hotmail.com (J.A.d.C.); amrodrigues.amr@gmail.com (A.M.R.); 3 Center for Research in Medical Mycology, Department of Pathology, Federal University of Espírito Santo (UFES), Vitoria 29043900, Brazil; 4 Department of Infectious and Parasitic Diseases, The Clinical Hospital of the Faculty of Medicine of the University of São Paulo, São Paulo 05403010, Brazil; wdsonluis@hotmail.com; 5 Department of Medical Clinic, Cassiano Antônio Moraes University Hospital (HUCAM), Federal University of Espírito Santo (UFES), Vitoria 29043900, Brazil; falqueto@npd.ufes.br

**Keywords:** *Sporothrix*, *Sporothrix brasiliensis*, sporotrichosis, genetic diversity, population structure, mating-type, antifungal susceptibility test, AFLP

## Abstract

Sporotrichosis is a neglected subcutaneous fungal infection that affects humans and animals worldwide caused by species belonging to the genus *Sporothrix*. This study aims to examine the range of genetic variations, assess molecular epidemiology significance, and explore potential modes of transmission of the *Sporothrix* species associated with the current sporotrichosis outbreaks in Espírito Santo, Brazil. In this investigation, 262 samples were evaluated, including 142 from humans and 120 from felines, collected between 2016 and 2021. The isolates were identified based on morphological and molecular characteristics. Sexual idiomorphs were determined by mating-type PCR using primers specific to the *MAT1-1* and *MAT1-2* loci. Amplified fragment length polymorphism (AFLP) was employed to assess the genetic variability of *Sporothrix* spp. Finally, antifungal susceptibility testing was performed following the CLSI M38-A2 protocol. Of the 142 human samples, 125 were identified as *S. brasiliensis* and 17 as *S. schenckii s. str.* The presence of *S. brasiliensis* was overwhelming (100%) during outbreaks, highlighting the significant role of domestic cats in the emergence of this species. Heterothallism was the only observed mating strategy. However, the *MAT1-2* idiomorph was predominant in cases of cat-transmitted sporotrichosis (χ^2^ = 202.976; *p* < 0.0001). Our AFLP results show significant intraspecific variability observed among *S. brasiliensis* isolates in Espírito Santo. Different genotypes forming subgroups within the same population suggest that these isolates do not originate from a single ancestor, indicating multiple emergences. Furthermore, terbinafine was the antifungal with the best results in vitro. However, in clinical practice, itraconazole remains the primary treatment choice. Sporotrichosis continues to advance in the state; therefore, the health system must outline one-health strategies to contain the disease to prevent future epidemics.

## 1. Introduction

Sporotrichosis is a subcutaneous mycosis caused by *Sporothrix* species, with a global distribution, particularly in tropical and subtropical regions [1,2]. The classical transmission route involves traumatic inoculation into the host’s subcutaneous tissue through contact with contaminated organic matter, soil, or thorny plants [3]. Additionally, the infection can occur through animal–animal or zoonotic interaction, primarily associated with scratches or bites from infected cats [4].

So far, 53 species belonging to the genus *Sporothrix* have been described [5]. Phylogenetic analyses have allowed the division of the genus into two clusters. The first cluster is the clinical or pathogenic clade, which includes species of significant clinical relevance, such as *S. brasiliensis*, *S. globosa*, *S. schenckii sensu stricto*, and *S. luriei*. The second cluster is the environmental clade, which encompasses other species that are generally non-pathogenic, with rare cases of infections in mammals [6]. *Sporothrix brasiliensis* is an emerging species in Brazil, reaching epidemic proportions and exhibiting significant pathogenicity for humans and animals [3,7].

Due to the endemic nature of the disease in Brazil, new cases of cat-transmitted sporotrichosis are emerging in South American countries, as already reported in Argentina, Chile, and Paraguay [8,9,10,11]. In Espírito Santo, the first human sporotrichosis via sapronotic was documented in 1982. For over three decades, the disease remained confined to rural and mountainous areas of the state [12]. However, from 2015 onwards, a new scenario of human sporotrichosis has been observed, characterized by the rapid spread and expansion of the disease into urban areas [13].

At the same time, feline sporotrichosis has been rapidly spreading throughout the state, primarily affecting intact male cats with outdoor habits in urban areas [14]. In this new epidemiological context, the infection is transmitted through a zoonotic route from cats to humans, with *S. brasiliensis* being the predominant species [13]. Sporotrichosis can be diagnosed through clinical–epidemiological manifestations and various laboratory tests, including the direct mycological examination (DME), culture, histopathology, cytopathology, molecular detection, and serology [15].

Currently, advances in molecular research of the genus *Sporothrix* have provided valuable insights into identifying and recognizing new and cryptic species within the pathogenic/clinical clades, enabling a better understanding of the genus taxonomy and biology [16]. In addition, various molecular tools have been developed to investigate the epidemiology of sporotrichosis expansion, including Amplified Fragment Length Polymorphism (AFLP) and simple sequence repeats (SSRs), which are considered DNA fingerprinting techniques [15,17,18,19].

Medically relevant *Sporothrix* species have traditionally been considered asexual due to the absence of morphological evidence of sexual development [20]. However, genomic studies have indicated that *S. schenckii s. str.*, *S. brasiliensis*, and *S. globosa* are heterothallic and may retain the ability to reproduce sexually. This finding significantly affects these pathogens’ population genetic variability and evolution. In addition, identifying sexual idiomorphs is crucial for understanding the population structure and geographic distribution of different *Sporothrix* species. For instance, in the epicenter of the cat-transmitted epidemic in Rio de Janeiro, there is a predominance of *MAT1-2* idiomorphs, while in Rio Grande Sul, outbreaks are driven by *MAT1-1* idiomorphs [21].

The choice of drug for treating sporotrichosis is based on various factors, including the severity and location of the lesions, the patient’s clinical condition, evaluation of drug interactions, potential adverse events, systemic involvement, and the specific *Sporothrix* species involved [3,22,23]. Itraconazole, potassium iodide, terbinafine, and amphotericin B are available in Brazil to treat sporotrichosis, with itraconazole being the preferred choice as the first-line antifungal [2]. Furthermore, antifungal sensitivity tests play a crucial role in the epidemiological surveillance of resistance, enabling the identification of potential therapeutic failures in the future [24].

This study aimed to investigate the genetic diversity of *Sporothrix* species obtained from human and animal samples and to gain a deeper understanding of the molecular epidemiology of sporotrichosis in Espírito Santo. This research also considered the unique morpho-physiological characteristics exhibited by these fungal pathogens.

## 2. Materials and Methods

### 2.1. Obtaining and Characterizing Isolates

In this investigation, we included 262 isolates suggestive of *Sporothrix* species. The human isolates (*n* = 142) were recovered from patients attending the University Hospital Cassiano Antônio Moraes (HUCAM), Federal University of Espírito Santo (UFES), between 2016 and 2021. At the same time, 120 *Sporothrix* isolates of veterinary origin were obtained from the stock culture collection at the Center for Research in Medical Mycology (CIMM), previously identified by Rediguieri et al. [14]. The patients with sporotrichosis did not have their animals evaluated for the disease, despite most reporting contact with animals suspected of sporotrichosis. Therefore, human and feline isolates were recovered based on the demand of the hospital (HUCAM), veterinary clinics, and zoonosis centers.

### 2.2. Data Collection from Patients Included in the Study

The patients’ demographic data were collected from medical records, the classification of the clinical condition, the probable source of infection, and the history of chronic diseases. Furthermore, relevant factors were also investigated as the time between the first symptoms and the diagnosis of sporotrichosis and the treatment choice before and after diagnosis.

### 2.3. Morphological Identification of Sporothrix Isolates

The isolates were identified according to established methods described by Marimon et al. [25]. Morphological features were evaluated from cultures grown on Potato Dextrose Agar (PDA; Difco Laboratories, Detroit, MI, USA) at 25 °C for 14 days. Characteristics of vegetative and conidiogenous structures were analyzed in micromorphology (presence of hyphae, pigmentation, and arrangement of conidia) and macromorphology (colony diameter, texture, and color of colonies).

### 2.4. Molecular Assays

#### 2.4.1. Extraction of Genomic DNA

Genomic DNA was extracted from 14-day-old monoconidial colonies grown at 25 °C cultivated on Sabouraud Dextrose Agar (SDA) (Difco™ BD/Sparks, MD, USA). DNA extraction and purification were performed using the Fast DNA kit (MP Biomedicals, Vista, CA, USA), following the protocol described by Rodrigues et al. [26]. The DNA concentrations and purity were estimated using a NanoDrop 2000 spectrophotometer (ThermoFisher Scientific, Wilmington, DE, USA). DNA quality was assessed by measuring the optical density (OD) at 260 nm and 280 nm wavelengths and calculating the OD 260/280 ratio. Only samples with OD 260/280 ratios between 1.8 and 2.0 were included in subsequent analyses. The DNA was then diluted to a final concentration of 100 ng/μL and stored at −20 °C until further use. As described in previous studies, all isolates were identified at the species level using a species-specific PCR targeting the calmodulin-encoding gene. In addition, reference strains representing the major phylogenetic groups in *Sporothrix* were included for detecting mating-type genes and genotyping using AFLP (Table 1).

#### 2.4.2. Species-Specific PCR Targeting the Calmodulin-Encoding Gene (*CAL*)

As previously described, the species-specific PCR assay was used to characterize isolates at the species level by targeting the *CAL* gene [26]. As a positive control, reference strains of *S. brasiliensis* (Ss54) and *S. schenckii s. str.* (Ss126) were included in PCR analysis. The PCR reactions were performed in a final volume of 25 μL, including 12.5 μL of PCR Master Mix buffer (2×) containing 3 mM MgCl_2_, 400 mM each dNTP, and 50 U/mL of Taq Polymerase (Promega Corporation, Madison, WI, USA). Additionally, 1 μL each of the forward and reverse species-specific primers were added (Sbra-F 5′ CCC CGT TTT GAC GCT TGG 3′ and Sbra-R 5′ CCC GGA TAA CCG TGT GTC ATA AT 3′, amplicon size 469 bp, or Ssch-F 5′ TTT CGA ATG CGT TCG GCT GG 3′ and Ssch-R 5′ CTC CAG ATC ACC GTG TCA 3′, amplicon size 331 bp) (Integrated DNA Technologies, San Diego, CA, USA). The remaining 8.5 μL of the reaction mixture consisted of water. An internal positive control (IPC) was included in the PCR to verify the presence of fungal DNA. The IPC primers targeted the 5.8 S region of the ribosomal DNA operon, generating a 101 bp amplicon in all species. To incorporate the IPC, 0.5 μL of each IPC primer (IPC-F 5′ATG CGATAC GTA ATG TGA ATT GC 3′ and IPC-R 5′ GAC GCT CGG ACA GGC ATG 3′) and 1 μL of the target DNA were added to the PCR reaction. A negative control reaction without *Sporothrix* DNA was included [26]. Electrophoresis was performed using a 1.2% agarose gel containing GelRed (Biotium, Hayward, CA, USA). The PCR products were loaded onto the gel and subjected to an electric current of 100 volts for one hour. The bands were visualized using UV light equipment L-Pix (Loccus Biotecnologia, São Paulo, Brazil).

#### 2.4.3. Duplex PCR for Identifying Mating-Type (*MAT*) Idiomorph

To assess the population structure and distribution of *Sporothrix* species, we analyzed feline and human isolates obtained and previously identified at the species level. Furthermore, data regarding the geographical origin of sporotrichosis cases were collected.

The duplex PCR assay was performed to detect the mating-type (*MAT*) gene in feline (*n* = 120) and human (*n* = 142) isolates. For this purpose, a set of primers was used, with one targeting the *MAT1-1* idiomorph (673 bp) and the other targeting the *MAT1-2* idiomorph (291 bp), following the method described by de Carvalho et al. [21]. Reference strains representing each idiomorph (*MAT1-1*: Ss05 and *MAT1-2*: Ss37) were included as positive controls, while a reaction without *Sporothrix* DNA served as a negative control. PCRs were performed in a final volume of 25 μL, which had 12.5 μL of PCR Master Mix buffer (2×; Promega Corporation, Madison, WI, USA), 9.6 μL of water, 0.6 μL each of forward and reverse primers (MAT1-1-1F 5′ GAT CCC TAC AAA AGC AAA TGG ACC ATG 3′; MAT1-1-1R 5′ CTG CAA TTG GGT TGT GCC TGA TG 3′; MAT1-2F 5′ CCA ATT TCC TCT TCC ACT ATT CGT CGC 3′; and MAT1-2R 5′ GCT TGA TAT CCA CGG CCA TCT TG 3′), and 1 μL of target DNA. The cycling conditions were as follows: 4 min at 94 °C, followed by 35 cycles of 1 min at 94 °C, 1 min at 62 °C, and 1 min at 72 °C. The reaction was concluded with a final step of 10 min at 72 °C. The amplicons were visualized on agarose gels using the previously described method.

Genotyping *Sporothrix* species by Amplified Fragment Length Polymorphism (AFLP).

To assess the inter- and intra-specific variability among *Sporothrix* strains, 110 isolates were randomly selected, including 100 isolates of *S. brasiliensis* and 10 isolates of *S. schenckii s. str.* Among the *S. brasiliensis* strains, 23 isolates obtained from feline sources were included, representing six cities in Espírito Santo. In addition to comparing these isolates, three clinically relevant species, namely *S. brasiliensis* (*n* = 15), *S. schenckii s. str.* (*n* = 6), and *S. globosa* (*n* = 6), were included as reference strains (Table 1).

This technique was performed following the protocols described by Vos et al. [27] and adapted by de Carvalho et al. [18]. It involved four steps: (i) restriction/ligation of the genomic DNA; (ii) pre-selective PCR; (iii) selective PCR; (iv) capillary electrophoresis. In summary, 200 ng of *Sporothrix* genomic DNA was digested using a combination of the two restriction enzymes EcoRI and MseI (New England Biolabs, Ipswich, MA) and ligated to EcoRI and MseI adapters simultaneously. Subsequently, a pre-selective amplification was performed using the primers EcoRI 5’ GAC TGC GTA CCA ATT C 3’ and MseI 5’ GAT GAG TCC TGA GTA A 3’ [18]. 

In the selective PCR, one microliter of the product was used for PCR amplification. The primers used were the same as in the previous step, adding the FAM fluorophore (blue) at the 5′ end of the EcoRI primer and two nucleotides at the 3′ end of each primer. The specific combination of nucleotides used was #5 (EcoRI-FAM-GA/MseI-AG), as previously described by Carvalho et al. [18]. All oligonucleotides were supplied by Integrated DNA Technologies (IDT, San Diego, CA, USA). PCR-amplified AFLP fragments were analyzed by capillary electrophoresis with a Seqstudio Genetic Analyzer alongside a LIZ600 internal size standard (Applied Biosystems. Foster City, CA, USA). 

First, the raw data were imported into BioNumerics v7.6 software (Applied Maths, Sint-Martens-Latem, Belgium). Next, we automated the selection of the amplified restriction products, focusing only on robust and high-quality fragments for further evaluation. Each electropherogram was carefully examined to eliminate low-confidence peaks such as stutter peaks, artifacts, pull-up peaks, and plus-adenine peaks to minimize scoring errors. A minimum threshold of 100 relative fluorescent units was set, and only peaks ranging in size from 50 to 500 base pairs were considered for analysis. They were then transformed into a binary data matrix in BioNumerics v7.6, where the presence of a fragment was represented as 1 and its absence as 0.

As recommended for dominant anonymous markers, the relationships between *Sporothrix* specimens and taxa were assessed using distance-based methods. The Jaccard similarity coefficient (JS) was calculated using the “fuzzy logic” option in BioNumerics v7.6 to determine pairwise genetic distances. Dendrograms were constructed using the unweighted pair-group method with arithmetic averages (UPGMA). The cophenetic correlation coefficient and standard deviation were used to evaluate the support and consistency of the clusters obtained through branch resampling.

#### 2.4.4. Dimensioning Analysis

Dimensionality reduction techniques were employed to generate three-dimensional plots, allowing the distribution of isolates based on their similarity. The minimum spanning tree (MST) model was utilized to illustrate the genetic relationships among the analyzed strains and to trace the transmission history of *S. brasiliensis*. In a given set of distances between n entries, the minimum spanning tree represents the tree with the minimum total branch length. The MSTs were constructed using BioNumerics v7.6 [19,28].

Principal component analysis (PCA) and multidimensional scaling (MDS) were also utilized in the investigation. PCA examines the dataset rather than the similarity matrix to characterize individuals and populations, enabling conclusions regarding origins, evolution, dispersion, and relatedness. It generates three-dimensional (3D) graphs. On the other hand, MDS provides an enhanced 3D graph based on the similarity matrix. Automated band matching was performed for all fingerprint entries in the comparison, considering minimum profiling of 5%, with optimization and position tolerances for band selection set to 0.10%. Default settings in BioNumerics v7.6 were applied for PCA and MDS, with the average subtracted for characters.

In addition, the self-organizing map (SOM), which is an unsupervised machine learning algorithm used for dimensionality reduction and data visualization, was employed to represent high-dimensional AFLP data in a lower-dimensional space (2D) based on their similarity [29,30]. All figures were exported and processed using Corel Draw X8 (Corel, Ottawa, ON, Canada). 

### 2.5. In Vitro Antifungal Susceptibility Profile

Antifungal susceptibility testing followed the Clinical and Laboratory Standards Institute (CLSI) M38-A2 protocol [31]. Pure powder forms of the tested drugs were provided by the manufacturers, including itraconazole (Janssen Pharmaceutica, Beerse, Belgium), posaconazole (Schering-Plough Incorporated, Kenilworth, NJ, USA), terbinafine (Sigma-Aldrich), and amphotericin B (Sigma Chemical Corporation, St. Louis, MO, USA). The antifungal agents were typically used at final concentrations ranging from 0.03 to 16 μg/mL. The fungal inoculum was prepared from *Sporothrix* isolates cultured on PDA and incubated at 25 °C for 14 days. The conidia were resuspended in a 10 mL sterile tube using sterile 0.85% saline. The suspension was mixed by vortexing for 20 s. The density of the suspension was adjusted to an optical density (OD) of 0.09–0.13, measured at 530 nm. This suspension was then diluted 1:50 in RPMI 1640 medium (Vitrocell, Campinas, São Paulo, Brazil) buffered to pH 7.0 with 0.165 mol/L of morpholino propane sulfonic acid (Sigma-Aldrich, St. Louis, MO, USA).

Aliquots of 100 µL of culture preparations in RPMI 1640 were inoculated into the flat-bottom wells of 96-well microtiter plates containing 100 µL the different concentrations of the tested antifungals. The final inoculum concentration ranged from 0.4 × 10^4^ to 5 × 10^4^ CFU/mL. *Aspergillus flavus* ATCC 204304 was included in each assay to verify the accurate dilution of the tested antifungals.

The minimum inhibitory concentration (MIC) was determined after 48 h of plate incubation at 37 °C by visually observing the plates using an inverted mirror. The MIC was defined as the lowest concentration that completely inhibited growth (100% inhibition) compared to the development controls for amphotericin, itraconazole, and posaconazole. For terbinafine, the MIC was defined as the lowest concentration that caused 80% growth inhibition. 

This study did not categorize the strains as wild type (WT) or non-WT. Thus, we only calculated the MIC distributions, range, MIC_50_, and MIC_90_ for all drugs tested. All tests were conducted in duplicate.

## 3. Results

### 3.1. Species and Mating-Type Idiomorph Identification

Through the macro and micro morphological characteristics of all isolates studied, it was possible to achieve a generic identification due to marked phenotypic overlap. A total of 262 isolates (142 from humans and 120 from felines) were evaluated using species-specific PCR. Among the isolates from human samples, 125 (88%) were identified as *S. brasiliensis*, and 17 were identified as *S. schenckii sensu stricto* (12%). On the other hand, all isolates from cats (*n* = 120, 100%) were exclusively identified as *S. brasiliensis*, indicating that it is the only agent responsible for feline sporotrichosis in Espírito Santo to date.

Our dataset’s only observed mating strategy was heterothallism, as both sexual idiomorphs (*MAT1-1* and *MAT1-2*) were detected. In feline isolates, a skewed distribution of *MAT1-1*:*MAT1-2* was observed (χ^2^ = 116.033; *p* < 0.0001), with 119 samples positive for *MAT1-2* (99.2%) and only one positive for *MAT1-1* (0.8%). A similar trend was observed in *S. brasiliensis* isolates from humans (*n* = 125; χ^2^ = 88.200; *p* < 0.0001), with 10 isolates (8%) having the *MAT1-1* idiomorph and 115 strains (92%) amplified for *MAT1-2*. In isolates identified as *S. schenckii s. str.* (*n* = 17), a balanced distribution of *MAT1*-*1*:*MAT1-2* was found (5:12; χ^2^ = 2.882; *p* = 0.0896), suggesting sexual reproduction.

### 3.2. Distribution Geographical of Sporothrix spp. concerning Mating-Type Idiomorph and Genetic Diversity

**Figure 1:**
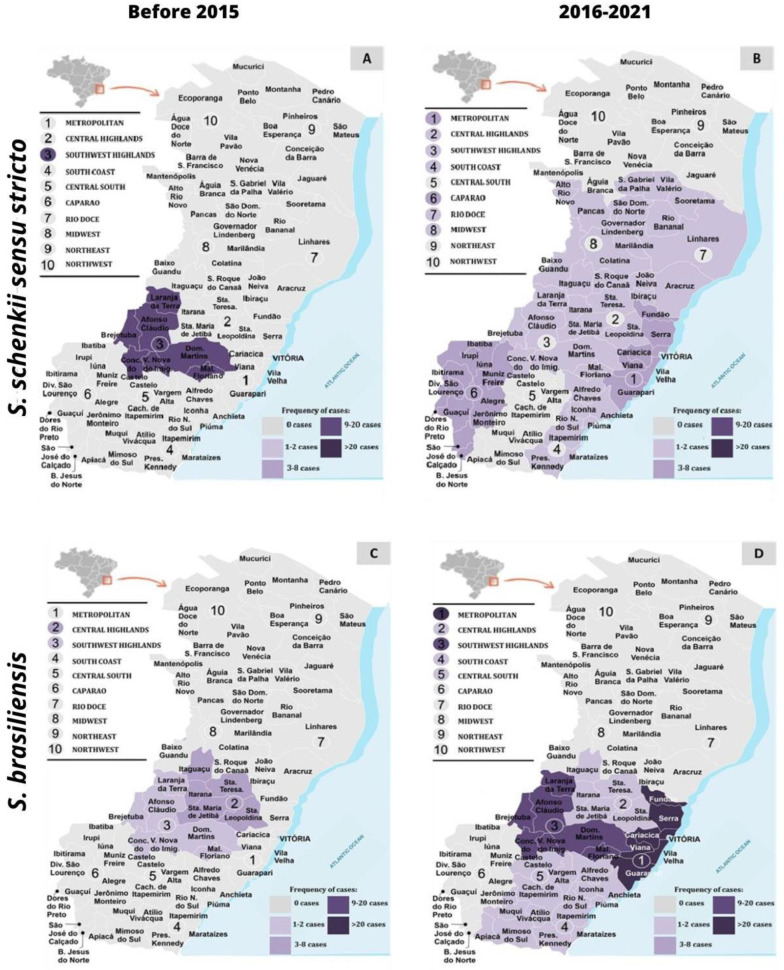
Progress of human sporotrichosis and distribution of *S. brasiliensis* and *S. schenckii sensu stricto* before 2015 [12] and between 2016 and 2021 in Espírito Santo, Brazil.

Out of the 142 cases of human sporotrichosis, we obtained epidemiological data from 132 patients. These cases were reported from ten different regions of the state: Metropolitan, Central Highland, Southwest Highland, South Coast, Central South, Caparao, Rio Doce, Midwest, Northeast, and Northwest (Figure 1).

According to the results, *S. brasiliensis* was the most prevalent species in the metropolitan region, accounting for 98 out of 125 cases (78.4%). On the other hand, *Sporothrix schenckii s. str.* was predominantly detected in the highland region and rural areas, as shown in Figure 1. It is important to note that before 2015 [13], sporotrichosis cases were mainly concentrated in these latter regions, with *S. schenckii* identified as the causative agent.

**Figure 2:**
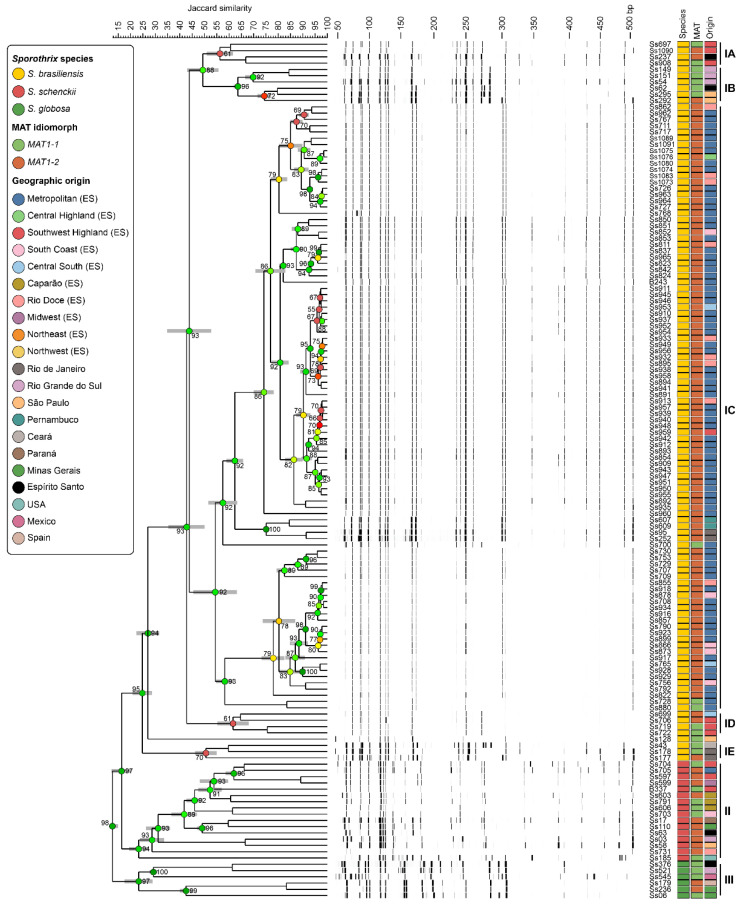
Mating-type idiomorph and genetic diversity of *Sporothrix* species causing sporotrichosis in Espírito Santo, Brazil. The dendrogram depicts the cluster profile generated by the AFLP method. The dataset consisted of 115 *S. brasiliensis* isolates (87 humans, 23 cats, and 15 reference strains), 16 *S. schenckii s. str.* isolates (10 human and 6 reference strains), and 6 *S. globosa* isolates (6 reference strains). The dendrograms were constructed using the Jaccard similarity coefficient and UPGMA clustering algorithm in BioNumerics software version 7.6.

A typical AFLP dendrogram based on Jaccard’s similarity coefficient is in Figure 2. Following the AFLP results, three major groups could be observed: (i) Group I (JS = 24.76% ± 3.39%), consisting of both reference and clinical strains of *S. brasiliensis*; (ii) Group II (JS = 23.25% ± 3.31%), comprising reference strains and clinical isolates of *S. schenckii s. str.*; (iii) Group III (JS = 23.24% ± 5.14%), formed by *S. globosa* strains, confirms species identification achieved through species-specific PCR. The dendrogram shows high cophenetic correlation coefficient values (>90%) in most branches.

Furthermore, by observing the geographical origin of the sporotrichosis cases included in the study, we could analyze the distribution of the mating-type idiomorphs within the *Sporothrix* species in Espírito Santo (Figure 2). Within *S. brasiliensis*, five main subgroups were observed. The isolates of *S. brasiliensis* with the *MAT1-1* mating-type clustered in subgroup IA (JS = 56.30% ± 4.75%) and IB (JS = 63.56% ± 0.97%), which were located at the top of the dendrogram. In contrast, subgroup IC (JS = 54.35% ± 8.25%) predominantly consisted of isolates with the *MAT1-2* mating-type. Of note, the isolates with the *MAT1-1* locus were primarily obtained from patients residing in the state’s mountainous regions (Southwest Highland), specifically in subgroups ID (JS = 61.62% ± 5.79%) and IE (JS = 50.59% ± 3.77%). Hence, the isolates with the *MAT1-2* locus predominate in most areas, except the Southwest Highland and Caparao regions, irrespective of the species.

**Figure 3:**
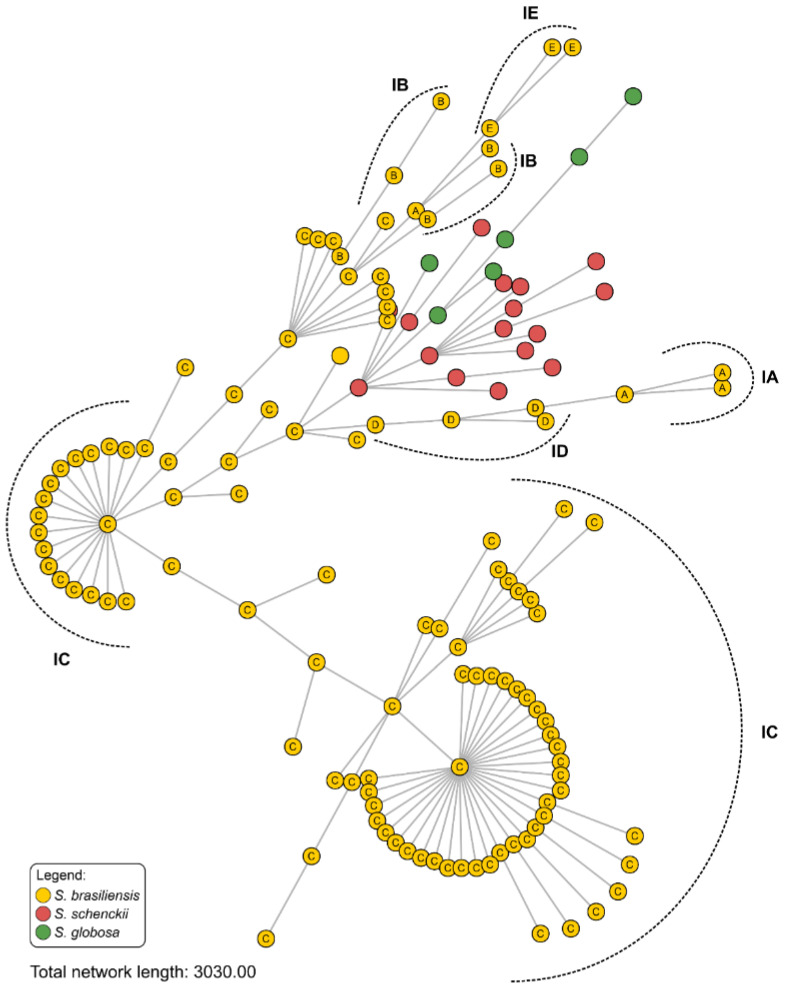
Minimum spanning tree (MST) derived from AFLP data of (i) 115 *S. brasiliensis* isolates (87 humans, 23 cats, and 15 reference strains), 16 *S. schenckii s. str.* isolates (10 humans and 6 reference strains), and 6 *S. globosa* isolates (6 reference strains). The dotted lines (IA-IE) represent the cluster profile obtained from the AFLP method and visualized in the dendrogram of Figure 2. The analysis was performed using BioNumerics v7.6 software.

**Figure 4:**
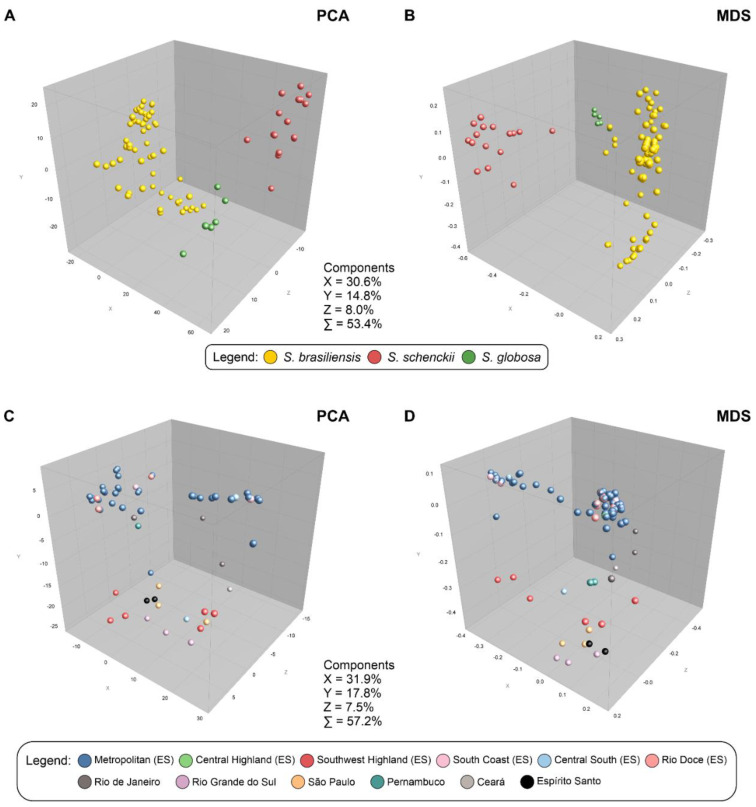
Principal component analysis (PCA-**A**) and multidimensional scaling (MDS-**B**) of 115 *S. brasiliensis* isolates (87 humans, 23 cats, and 15 reference strains), 16 *S. schenckii s. str.* isolates (10 humans and 6 reference strains), and 6 *S. globosa* isolates (6 reference strains). The isolates were plotted in three-dimensional space and colored according to the genetic groups. *Sporothrix brasiliensis* isolates (*n* = 115) were analyzed separately (**C**,**D**) and colored according to geographic origin. The analysis was performed using the BioNumerics v7.6 software.

Cluster analysis performed using the MSTs (Figure 3) identified evidence of genotype dispersion across Espírito Santo, where the main genetic groups circulating in recent cat-transmitted outbreaks share relationships with *S. brasiliensis* genotypes occurring in the neighboring state of Rio de Janeiro. Next, population structure was evaluated using multivariate analysis such as principal component analysis (PCA) and multidimensional scaling (MDS) (Figure 4). A robust population structure was identified using PCA and MDS, with clear interspecific separation. In this scenario, the proximity between Espírito Santo and Rio de Janeiro genotypes is evident in both analyses. The three main components (X, Y, and Z) explain 53.4% of the diversity in recent outbreaks (Figure 4).

**Figure 5:**
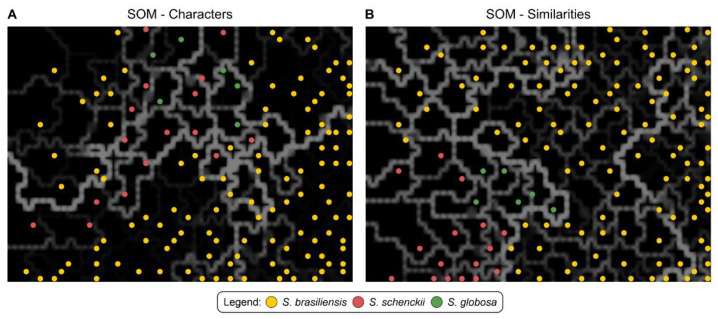
Distribution of *Sporothrix* spp. Genotypes generated by AFLP using a Self-Organizing Map (SOM). Kohonen maps using (**A**) character data or (**B**) the similarity matrix. A total of 115 *S. brasiliensis* isolates (87 humans, 23 cats, and 15 reference strains), 16 *S. schenckii s. str.* isolates (10 humans and 6 reference strains), and 6 *S. globosa* isolates (6 reference strains) were included in this study. The dimensioning analyses were performed using BioNumerics v7.6 to assess the consistency of the differentiation of the populations defined by the cluster analysis. The distance between black blocks can be inferred by observing the thickness and brightness of the lines (white, grey) connecting them. The thicker and lighter the line, the greater the distance between the samples in the black blocks and their neighboring blocks. Isolates were assigned specific colors corresponding to their genetic groups.

Figure 5 shows the dimensional reduction analysis of AFLP data to reconstruct the epidemiological scenario in Espírito Santo. Judging from neural networks, we observed the presence of reconstructed scenarios with *S. brasiliensis* genotypes displaying little genetic diversity, characteristic of outbreaks with a common source, as classically occurs during events of cat-transmitted sporotrichosis. These scenarios are depicted by genotypes separated by thin lines of colors ranging from dark gray to black (subgroup IC) in Kohonen’s maps. In contrast, we also observed epidemiological scenarios where the *S. brasiliensis* isolates were separated by thick lines and displayed colors closer to white (subgroups IA, IB, ID, and IE), indicating significant genetic distance. This variation may be attributed to the species’ intrinsic diversity and multiple introductions in Espírito Santo.

### 3.3. Clinical and Epidemiological Characteristics of Human Sporotrichosis

**Figure 6:**
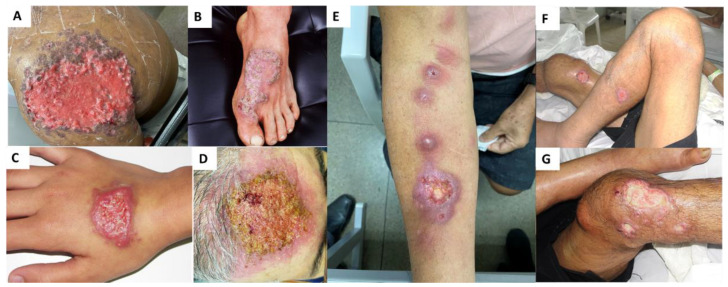
Clinical manifestations of human sporotrichosis: (**A**–**D**) Localized sporotrichosis: (**A**) Left shoulder injury; (**B**) Left foot injury; (**C**) Right-hand injury; (**D**) Head injury; (**E**) Cutaneous-lymphatic sporotrichosis in the right arm; (**F**,**G**) Systemic sporotrichosis.

Concerning the patients included in this investigation, as shown the Table 2, the majority were female (*n* = 79/59.9%) with a mean/median age of 40/41 years, respectively. The primary clinical manifestation was the lymphocutaneous form (*n* = 92/69.7%), followed by the cutaneous-fixed form (*n* = 37/28%) and, to a lesser number, the disseminated form (*n* = 3/2.3%), which is the most severe form of the disease. Unfortunately, one immunosuppressed patient (HIV/AIDS) in the study succumbed to the disease. Figure 6 illustrates the clinical manifestations of sporotrichosis observed in patients treated at HUCAM.

Regarding disease transmission, 84.9% (*n* = 112) of the patients reported previous contact with sick felines, thus characterizing the route of zoonotic transmission as the most frequent in the state in recent years (Table 2). Among the patients, 77 (58.3%) did not present any chronic or underlying disease, indicating that they were immunocompetent. Among the remaining patients (*n* = 55/41.7%), the main comorbidities reported were systemic arterial hypertension (*n* = 25/18.9%), diabetes mellitus (*n* = 8/6%), and human immunodeficiency virus infection (*n* = 2/1.5%).

**Figure 7:**
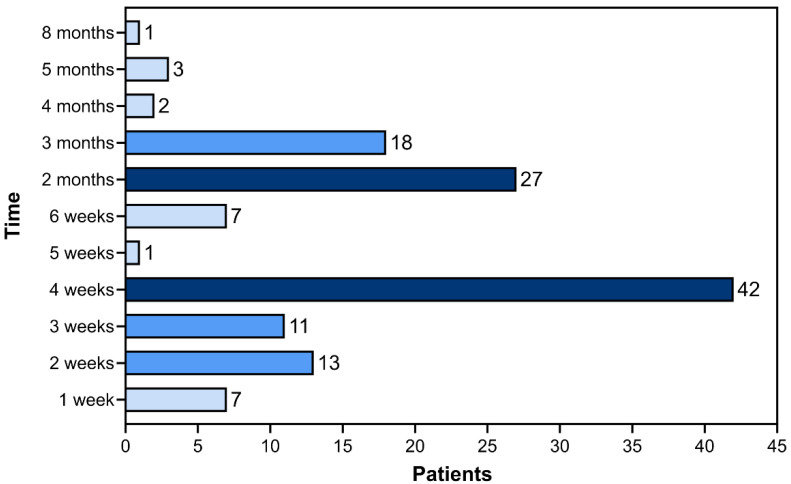
Time divergence between the onset of the first symptoms of sporotrichosis and the definitive diagnosis in the study patients (*n* = 132). The intensity of the graph color increases proportionally with the number of patients, categorized into intervals of 0 < 10 cases, 10 < 20 cases, and >20 cases.

A wide variation was observed between the onset of the first symptoms and the definitive diagnosis, ranging from 1 week to 8 months (Figure 7). Most patients (*n* = 77/58.3%) were diagnosed between 4 and 8 weeks from the appearance of initial signs and symptoms. The diagnosis time of 4 weeks was the most common among the other observed intervals (*n* = 42/31.8%). Figure 7 illustrates the time difference between the first symptoms and the definitive diagnosis of human sporotrichosis.

Before the definitive diagnosis of sporotrichosis, the treatment was generally based on antibiotics and combination therapy, primarily involving broad-spectrum antibiotics. In a few cases (*n* = 16, 12.1%), combined therapy was used with antifungal agents. Other medications, such as antiparasitics and antivirals, were also administered. Table 3 describes the drugs utilized.

After receiving a definitive diagnosis, therapeutic management was determined based on the characteristics and severity of the lesions (Table 3). Itraconazole was the most commonly used antifungal agent (*n* = 98/74.2%), followed by potassium iodide (*n* = 21/15.9%). Amphotericin B was only administered in one case (0.8%). Combination therapies included itraconazole with potassium iodide (*n* = 10/7.6%) and amphotericin B with itraconazole (*n* = 2/1.5%).

## 4. In Vitro Antifungal Susceptibilities

The 116 isolates included in inter and intraspecific analysis by AFLP were also evaluated for their in vitro antifungal susceptibility profile (96 *S. brasiliensis* isolates, 20 from cats and 84 humans, and 12 *S. schenckii* isolates). The MIC values for the reference strain used as control fell within the range established for the tested drugs: amphotericin B (0.5–4 μg/mL), itraconazole (0.25–0.5 μg/mL), posaconazole (0.06–0.5 μg/mL), and terbinafine (0.25–0.5 μg/mL). This validates the appropriate dilution of antifungals.

The MIC values determined in this study are described in Table 4 and Table 5. For isolates obtained from human samples, terbinafine was the most effective antifungal for both *S. brasiliensis* and *S. schenckii s. str.*, with MIC_90_ values of 0.125 μg/mL. Among the azoles, posaconazole demonstrated high MIC values for *S. schenckii s. str.* (MIC_90_ = 2.0 μg/mL), while the isolates of *S. brasiliensis* had MIC_90_ values twice as high as those of *S. schenckii s. str.* for posaconazole. For itraconazole and amphotericin B, the MIC_90_ values for both species were the same, 4.0 μg/mL and 2.0 μg/mL, respectively (Table 4).

Regarding isolates of *S. brasiliensis* recovered from feline samples, terbinafine was also the most effective antifungal, with MIC_90_ values of 0.125 μg/mL, followed by amphotericin B and posaconazole (MIC_90_ = 2.0 μg/mL). However, these isolates showed higher MIC_90_ values for itraconazole, with an MIC_90_ of 4.0 μg/mL (Table 4).

## 5. Discussion

In Brazil, human sporotrichosis occurs in 25 out of 26 Brazilian states [3,32]. Since 1997, the zoonotic transmission pattern has been the most notable in sporotrichosis, affecting families with unfavorable sanitary and socioeconomic conditions and veterinary professionals and assistants. The primary source of infection is through cats (*Felis catus*), with transmission occurring through bites, scratches, and contact with exudate from cutaneous-mucosal and respiratory lesions [33,34,35]. Espírito Santo is located in Southeastern Brazil, a region of high endemicity for cat and human sporotrichosis, particularly in Rio de Janeiro. This region encompasses semi-arid, tropical, high-altitude tropical or subtropical, humid coastal, and maritime temperate climates, all providing suitable conditions for developing *Sporothrix* species in the pathogenic clade [12].

We reconstructed the epidemiological scenario of cat-transmitted sporotrichosis in Espírito Santo based on clinical–laboratory data, molecular biology, and bioinformatics analyses. Our findings indicate the emergence of sporotrichosis, with the involvement of multiple genotypes of *S. brasiliensis* that exhibit significant similarities to those circulating during the long-lasting epidemic in Rio de Janeiro.

The laboratory diagnosis of sporotrichosis in routine clinical practice is typically carried out through microorganism culture, followed by identifying the etiological agent based on morpho-physiological characteristics. However, the slow growth of cultures and the significant phenotypic similarities among closely related species can challenge identification [15,19,22]. For example, *S. brasiliensis* and *S. schenckii* bear single-celled hyaline sympodial conidia, obovoidal (2–6 × 1–4 µm) and clustered in the apical part of the conidiophores, resembling a “daisy” appearance. The secondary conidia are observed along undifferentiated hyphae and in *S. schenckii s. str.*, they are typically triangular [25], whereas, in *S. brasiliensis,* sessile conidia are brown to dark brown, thick-walled, globose to subglobose (2.5–5 × 2–3 µm) [25]. Furthermore, *S. brasiliensis* exhibits different phenotypes: one phenotype referred to as “albino,” in which melanin production is not observed, and another melanized phenotype, which is characteristic of the genus [9]. However, as observed in our study, these characteristics can be easily confused, making morphological characterization an inadequate tool for accurately identifying species [13,14]. Therefore, morphology only enables identification at the genus level and may provide presumptive speciation in a few cases [36].

In our study, we recovered and identified only one albino isolate, which was morphologically and molecularly confirmed as *S. brasiliensis*. The patient with this isolate presented with lymphocutaneous sporotrichosis and was successfully treated with itraconazole. The patient experienced a favorable clinical outcome without any complications. Oliveira et al. [37] described a case in which two genetically and phenotypically distinct *S. brasiliensis* strains were isolated from a single patient lesion. These isolates exhibited variable melanin production. Surprisingly, in the murine model, the albino phenotype demonstrated higher virulence than the melanized isolate. The albino phenotype caused a more severe form of sporotrichosis, characterized by elevated levels of inflammatory infiltrates and lesions in all organs, observed in histopathological findings. It is worth noting that although melanin is an important virulence factor, it is not the sole determinant for predicting virulence [37].

In our study, molecular identification using species-specific PCR was employed as the gold standard for identifying medically relevant *Sporothrix* species. This approach confirmed the presence of both *S. schenckii s. str.* and *S. brasiliensis* as the causative agents of human and feline sporotrichosis in Espírito Santo. These findings indicate that two distinct transmission routes exist in this region. The sapronotic pathway is commonly associated with *S. schenckii s. str.* as the primary causative agent.

In Espírito Santo, cases of human sporotrichosis have been reported in the literature since 1982. These cases were predominantly found in rural and mountainous areas [12,38,39]. In a study evaluating medical records from 1982 to 2012, Caus et al. [12] diagnosed 171 cases of human sporotrichosis. Among these, 160 patients (93.57%) were confirmed through laboratory diagnosis by isolating *S. schenckii sensu lato*, while 11 cases (6.43%) were diagnosed based on the patient’s clinical characteristics. Additionally, in Espírito Santo, de Araujo et al. [38] identified 18 *Sporothrix* isolates at the molecular level. The most prevalent species was *S. schenckii sensu stricto* (66.7%), followed by *S. brasiliensis* (27.8%). In addition, these investigators identified *S. globosa* (5.5%), indicating the circulation of other *Sporothrix* species. Therefore, sporotrichosis presented in these studies was, until 2015, a disease restricted to rural and mountainous areas of the state, with slightly lower temperatures and altitudes when compared to the metropolitan region of the state, with transmission from the fungus in its saprophytic form.

According to our findings and the studies conducted by Caus et al. [12], Cruz Bahiense Rocha et al. [13], and Rediguieri et al. [14] showed a rapid spread of *S. brasiliensis* across the state between 2015 and 2021. It is worth noting that this series of cases included patients exclusively treated by the infectious diseases service at HUCAM, a reference center for sporotrichosis in the state.

In a recent study by da Cruz Bahiense Rocha and colleagues [13] analyzing 75 *Sporothrix* isolates, 76% were confirmed as *S. brasiliensis* and 24% as *S. schenckii sensu stricto*. This study revealed a new scenario of sporotrichosis in the state, characterized by the rapid spread and expansion of the disease in urban areas. The disease has now reached multiple municipalities, including those with climate profiles different from those observed by Caus et al. [12]. These new cases occur in locations with higher temperatures and higher humidity levels.

Our data revealed a strong association between *S. brasiliensis* and epizootic and zoonotic sporotrichosis transmission. The alternative transmission route has proven to be highly effective in spreading the disease, with 84.9% of patients reporting previous contact with diseased felines that tested positive for sporotrichosis. Additionally, our findings align with previous studies, showing that women are the most affected group [13,40]. In the population studied, no cases of sporotrichosis were found among veterinary professionals or animal caretakers. However, we must note that these professionals are also at risk of contracting the disease in our state (unpublished data). Our findings indicate the ongoing expansion of cat-transmitted sporotrichosis in urban areas of Espírito Santo since 2015, with no signs of mitigation.

Considering the influence of clonal amplification on mating-type frequencies, our results indicate that during outbreaks in the feline population, *S. brasiliensis* genotypes tend to be predominantly clonal. However, this does not necessarily mean the absence of sexual reproduction but instead suggests the emergence of a successful genotype. Mating-type distributions suggest heterothallism as the predominant mating strategy. From an epidemiological perspective, the frequency of *MAT1-1* isolates was significantly higher in *S. brasiliensis* from Rio Grande do Sul, while *MAT1-2* isolates were dominant in Rio de Janeiro [20,21,41]. Thus, *S. brasiliensis* isolates from Espírito Santo carry the *MAT1-2* locus and exhibit genetic similarities with the Rio de Janeiro clade, indicating a unidirectional migration from Rio de Janeiro. In contrast, previous studies have demonstrated a 1:1 ratio of *S. schenckii s. str.* isolates in São Paulo, Paraná, and Goiás, which is consistent with our findings in Espírito Santo [20].

Significant intraspecific variability among *S. brasiliensis* isolates in Espírito Santo was observed, with different genotypes divided into five subgroups within the same population. Particularly noteworthy are isolates belonging to the subgroup IC, corresponding to the Rio de Janeiro clade. Additionally, the isolates from the Southwest Highland and Central South regions, both mountainous areas, formed two distinct subgroups (IA and ID) that were separated from the other isolates.

This suggests the presence of a single wild focus in this region that is gradually spreading. Additionally, it is essential to emphasize that the mountainous region of Espírito Santo is characterized by a high-altitude tropical climate, with temperatures below 22 °C during the summer and cold winter. Rainfall is concentrated in the summer months. In contrast, the metropolitan area (coastal) has a tropical hot and rainy climate without a distinct cold season.

Hence, it is worth investigating how climatic conditions can influence the distribution of *Sporothrix*, as it is an important aspect that requires further exploration [15]. De Carvalho et al. [18] demonstrated a high genetic diversity among medically relevant *Sporothrix* species, indicating their ability to disperse and adapt to different environments worldwide. Moreover, these findings support the notion of disease progression and the expansion of various distinct genotypes of *S. brasiliensis* across the country, in agreement with the hypothesis that the state of Rio de Janeiro is likely the contemporary epicenter of the disease’s origin [17,42].

Regarding the patient profile of individuals affected by sporotrichosis transmitted by felines in Espírito Santo (ES), it has been observed that females are more commonly affected, and the lymphocutaneous form of the disease is frequently encountered [35,43,44,45]. Furthermore, the average time from the onset of signs and symptoms to diagnosis was eight months. A study by Caus et al. [12] examined sporotrichosis cases diagnosed at HUCAM over 30 years (1982–2012), the same hospital from which the patients in our study were derived. Among the male patients diagnosed during this period (80.7% of the total cases), most were engaged in activities involving direct contact with plants and soil, such as gardening and agriculture (68.4%).

The most common form of sporotrichosis observed in our study was the lymphocutaneous (*n* = 120/70.2%), followed by the fixed-cutaneous (*n* = 49/28.6%) and the extracutaneous (*n* = 2/1.2%) and the time elapsed from the appearance of the lesion to the definitive diagnosis of the patients varied between 15 days and 10 years. Therefore, sporotrichosis, previously associated with occupational and recreational activities, is now linked to contact with cats infected by *Sporothrix* spp. Particularly affected groups include children, older individuals, and women with low socioeconomic status [34,35].

This study highlights the shift in the clinical landscape of sporotrichosis in Espírito Santo with the emergence of cat-transmitted sporotrichosis. Despite the rapid increase in the prevalence of the disease in the state, the knowledge and information regarding sporotrichosis have lagged, as is often the case with neglected fungal infections.

Regarding treating sporotrichosis patients in this study, 12 cases experienced therapeutic failure when treated with itraconazole as monotherapy. As a result, combined therapy with potassium iodide was required in 11 of these cases. In a more severe case, amphotericin B was an alternative drug. It is worth noting that itraconazole has been the treatment of choice for sporotrichosis, and most published series have reported disease remission and clinical cure with its use [35,44,46]. In contrast, potassium iodide has been used as a treatment for sporotrichosis for over 100 years [47,48,49]. Nonetheless, it is associated with side effects, including metallic taste, nausea, vomiting, and loss of appetite. As a result, itraconazole has emerged as an alternative treatment for refractory cases. Currently, itraconazole is considered the first-line antifungal treatment for sporotrichosis [48,49]. In our study, potassium iodide was used as monotherapy in patients who did not respond to itraconazole, although itraconazole was the primary drug employed. According to the survey conducted in ES by Caus et al. [12], potassium iodide achieved therapeutic success in 97.7% of patients (*n* = 168/171), with itraconazole and terbinafine being utilized for the remaining cases until 2012.

In vitro susceptibility testing was conducted to assess antimicrobial resistance, and the results showed that among the 23 feline isolates, itraconazole exhibited the highest MIC values, followed by posaconazole, amphotericin B, and terbinafine. Terbinafine demonstrated the highest effectiveness, with MIC values ranging from 0.03 µg/mL to 0.125 µg/mL. Furthermore, no variation in the sensitivity profile was observed among different regions of Espírito Santo. According to the epidemiologic cutoff values (ECVs) established by Espinel–Ingroff et al. [50], most of our isolates were classified as wild type. Our study identified the highest MIC values (8 µg/mL) in human isolates: one from *S. schenckii s. str.* obtained in 2016 and two from *S. brasiliensis* acquired in 2018 and 2020. While ECVs cannot predict the clinical response to therapy, elevated MIC values suggest that isolates may not respond well to treatment with these agents. However, *S. brasiliensis* isolates were successfully treated with itraconazole without further complications. The *S. schenckii s. str.* isolate was effectively eliminated using potassium iodide as the first-choice treatment, resulting in therapeutic success.

Notably, resistance to itraconazole has already been described in all medically relevant *Sporothrix* species. In a study conducted by Vettorato et al. [51] in Porto Alegre, Brazil, a female patient who was infected by *S. schenckii s. str.* received itraconazole as the initial treatment choice. In the in vitro susceptibility testing, MIC values equal to 16.0 µg/mL were observed for this drug. However, therapy was not discontinued. After 48 days of treatment, the patient did not show good clinical progress. Consequently, itraconazole was substituted by terbinafine at a dose of 500 mg/day, as it exhibited satisfactory in vitro results (MIC = 0.25 µg/mL). After four months of treatment, the patient achieved a clinical cure with no disease recurrence. In the case of *S. globosa* infection through the sapronotic route in Minas Gerais, Brazil, the initial treatment choice of topical ketoconazole and itraconazole 200 mg/day over two years showed no efficacy or good clinical progress. However, after the correct identification of the agent, the initial therapy was replaced by potassium iodide for six months, resulting in a successful cure [52].

Regarding in vitro MICs values obtained in various investigations, they were also observed that terbinafine is the most effective drug [53,54,55,56]. Maschio–Lima et al. [55] evaluated 189 cases of feline sporotrichosis caused by *S. brasiliensis* in São José do Rio Preto, São Paulo. Terbinafine (MIC_50_= 0.25 µg/mL, MIC_90_= 1.0 µg/mL), ketoconazole (MIC_50_= 0.5 µg/mL, MIC_90_= 1.0 µg/mL), and itraconazole (MIC_50_= 0.5 µg/mL, MIC_90_= 1.0 µg/mL) demonstrated better antifungal activity in vitro. On the other hand, amphotericin B and potassium iodide were less effective, with higher MIC values (MIC_50_= 2.0 µg/mL, MIC_90_= 8.0 µg/mL, and MIC_50_= 125 mg/mL, MIC_90_= 250 mg/mL, respectively).

In another study conducted with 47 feline isolates in Rio de Janeiro, a hyperendemic area of sporotrichosis in Brazil, Machado [57] observed that among the azoles, ketoconazole showed MIC_50_ values equal to 0.5 µg/mL and MIC_90_ values equal to 1.0 µg/mL. In contrast, itraconazole showed higher MIC_50_ e MIC_90_ values (1.0 µg/mL and 2.0 µg/mL, respectively). Despite the lower MIC values of ketoconazole, it has shown a less satisfactory therapeutic response in practice than itraconazole. Amphotericin B presented MIC_50_ values of 1.0 µg/mL and MIC_90_ values of 2.0 µg/mL. Amphotericin B showed values of MIC_50_ = 1.0 µg/mL and MIC_90_ = 2.0 µg/mL. Until then, we had no susceptibility data to antifungals in the state for feline and human sporotrichosis. Therefore, these are the first results obtained from strains in Espírito Santo (ES).

## 6. Conclusions

The identification based on morphological criteria needs to be revised for differentiating the etiological agents of sporotrichosis, and molecular tools have proven to be more accurate in species identification. The emergence of cat-transmitted sporotrichosis in Espírito Santo, driven by *S. brasiliensis*, continues to spread without signs of mitigation. However, we observed a significant genetic diversity during epizootics and zoonosis, with a distinct subgroup showing genetic similarities to the Rio de Janeiro clade. Additionally, we identified an autochthonous area of *S. brasiliensis* in the mountainous regions that have not spread to other sites. In vitro, terbinafine demonstrated the highest effectiveness among antifungals, although its use in clinical practice is limited. The lack of awareness of the disease among the population and the absence of adequate control measures in Espírito Santo has increased the risk of reaching vulnerable people, leading to more severe cases and possible outbreaks. Overall, our study contributes to a better understanding of the disease’s history in the state and enhances scientific knowledge about the fungus’s biology in our environment.

## Figures and Tables

**Table 1:** Reference strains, species, source, origin, and mating-type of *Sporothrix* spp. isolates used for the detection of mating-type genes and genotyping by AFLP.

Isolate Code	Other Code	Species	*MAT*	Source	Geographic Origin
Ss43	-	*S. brasiliensis*	*1-1*	Human	Ceara-BR
Ss54	CBS 132990	*S. brasiliensis*	*1-1*	Cat	Rio Grande do Sul-BR
Ss62	CBS 132991	*S. brasiliensis*	*1-1*	Human	Espírito Santo-BR
Ss95	-	*S. brasiliensis*	*1-2*	Human	Rio de Janeiro-BR
Ss128	-	*S. brasiliensis*	*1-1*	Human	São Paulo-BR
Ss149	-	*S. brasiliensis*	*1-1*	Human	Rio Grande do Sul-BR
Ss151	CBS 132994	*S. brasiliensis*	*1-1*	Dog	Rio Grande do Sul-BR
Ss177	FMR 8309	*S. brasiliensis*	*1-2*	Human	Rio de Janeiro-BR
Ss178	CBS 120339	*S. brasiliensis*	*1-1*	Human	Rio de Janeiro-BR
Ss237	-	*S. brasiliensis*	*1-2*	Human	Espírito Santo-BR
Ss252	CBS 133011	*S. brasiliensis*	*1-2*	Cat	Rio de Janeiro-BR
Ss292	-	*S. brasiliensis*	*1-2*	Cat	São Paulo-BR
Ss295	-	*S. brasiliensis*	*1-1*	Cat	São Paulo-BR
Ss607	-	*S. brasiliensis*	*1-2*	Human	Pernambuco-BR
Ss609	-	*S. brasiliensis*	*1-2*	Human	Pernambuco-BR
Ss03	CBS 132963	*S. schenckii*	*1-2*	Human	Rio Grande do Sul-BR
Ss17	-	*S. schenckii*	*1-2*	Human	Paraná-BR
Ss58	-	*S. schenckii*	*1-2*	Human	São Paulo-BR
Ss63	CBS 132968	*S. schenckii*	*1-2*	Human	Espírito Santo-BR
Ss110	-	*S. schenckii*	*1-2*	Human	Minas Gerais-BR
Ss185	CBS 359.36	*S. schenckii*	*1-1*	Human	USA
Ss06	CBS 132922	*S. globosa*	*1-1*	Human	Minas Gerais-BR
Ss179	CBS 120340	*S. globosa*	*1-2*	Human	Spain
Ss236	CBS 132925	*S. globosa*	*1-2*	Human	Minas Gerais-BR
Ss376	-	*S. globosa*	*1-1*	-	Espírito Santo-BR
Ss521	-	*S. globosa*	*1-1*	Human	Rio Grande do Sul-BR
Ss545	-	*S. globosa*	*1-1*	-	Mexico

All “Ss” strains belong to the culture collection of the Federal University of São Paulo (UNIFESP), Paulista School of Medicine (EPM); CBS, culture collection of the Westerdijk Fungal Biodiversity Institute, Utrecht, The Netherlands; BR, Brazil. Mating-type (*MAT*) gene.

**Table 2:** Clinical and epidemiological characteristics of sporotrichosis patients included in this study (*n* = 132).

Patients		
Variables	N	Percentage (%)
Gender		
Female	79	59.9
Male	53	40.1
Clinical presentation		
Lymphocutaneous	92	69.7
Cutaneous-Fixed	37	28
Disseminated	3	2.3
Source of infection		
Zoonosis	112	84.9
Environmental	20	15.1

**Table 3:** Treatment regimens were administered to patients included in this study before and after diagnosing human sporotrichosis (*n* = 132).

Therapy of Choice before Diagnosis	* Number of Patients/%	Treatment of Choice after Diagnosis Definitive	Number of Patients/%
Cephalexin	43/32.6	Itraconazole	98/74.2
Amoxicillin + Clavulanate	19/14.4	Potassium iodide	21/15.9
Ceftriaxone	14/10.6	Amphotericin B	1/0.8
Penicillin G	13/9.9	Itraconazole + Potassium Iodide	10/7.6
Sulfamethoxazole + trimethoprim	13/9.9	Amphotericin B + Itraconazole	2/1.5
Amoxicillin	11/8.3		
Itraconazole	11/8.3		
Clindamycin	8/6.1		
Azithromycin	7/5.3		
Levofloxacin	7/5.3		
Ciprofloxacin	6/4.6		
Mupirocin	3/2.3		
Terbinafine	3/2.3		
Fluconazole	2/1.5		
Acyclovir	1/0.8		
Doxycycline	1/0.8		
Ivermectin	1/0.8		

* Some patients received multiple medications before the diagnosis.

**Table 4:** In vitro antifungal susceptibility profile of the 96 isolates of *Sporothrix* spp. from human samples against amphotericin B, itraconazole, posaconazole, and terbinafine.

Specie (*n*)	Antifungal	MIC Range µg/mL	MIC_50_	MIC_90_
*S. brasiliensis* (84)	Amphotericin B	0.5–2.0	2.0	2.0
	Itraconazole	0.5–8.0	2.0	4.0
	Posaconazole	1.0–4.0	2.0	4.0
	Terbinafine	0.03–0.25	0.06	0.125
*S. schenckii* (12)	Amphotericin B	1.0–2.0	2.0	2.0
	Itraconazole	1.0–8.0	2.0	4.0
	Posaconazole	1.0–2.0	2.0	2.0
	Terbinafine	0.03–0.125	0.06	0.125

MIC—Minimum inhibitory concentration; MIC_50_: MIC value capable of inhibiting 50% of the tested samples; MIC_90_: MIC value capable of inhibiting 90% of the tested samples.

**Table 5:** In vitro antifungal susceptibility profile of the 23 feline isolates against amphotericin B, itraconazole, posaconazole, and terbinafine.

Specie (*n*)	Antifungal	MIC Range µg/mL	MIC_50_	MIC_90_
*S. brasiliensis* (20)	Amphotericin B	0.5−2.0	1.0	2.0
	Itraconazole	1.0−4.0	2.0	4.0
	Posaconazole	0.5−2.0	2.0	2.0
	Terbinafine	0.03−0.125	0.06	0.125

MIC—Minimum inhibitory concentration; MIC_50_: MIC value capable of inhibiting 50% of the tested samples; MIC_90_: MIC value capable of inhibiting 90% of the tested samples.

## Data Availability

The data presented in this study are available within the article.
